# New insights in the interactions between African trypanosomes and tsetse flies

**DOI:** 10.3389/fcimb.2013.00063

**Published:** 2013-10-16

**Authors:** Jan Van Den Abbeele, Brice Rotureau

**Affiliations:** ^1^Unit of Veterinary Protozoology, Department of Biomedical Sciences, Institute of Tropical Medicine, Group ParasitologyAntwerpen, Belgium; ^2^Laboratory of Zoophysiology, Department of Physiology, University of GhentGhent, Belgium; ^3^Trypanosome Cell Biology Unit, Institut Pasteur, CNRS URA 2581Paris, France

**Keywords:** Trypanosoma, African trypanosomes, parasite cycle, tsetse fly, vector, infection, interaction

African trypanosomiases are vector-borne diseases of human and their livestock, with devastating socio-economical consequences for the Sub-Saharan African continent. These diseases are caused by flagellated unicellular parasites named African trypanosomes that are almost exclusively transmitted by the bite of tsetse flies. Here, both male and female tsetse flies are obligatory blood feeders able to carry and transmit trypanosomes during their entire life span.

Two species of African trypanosomes are responsible for Human African Trypanosomiasis (HAT), also known as sleeping sickness: *Trypanosoma brucei gambiense* in Central/West Africa and *T. b. rhodesiense* in East/Southern Africa. The *T. b. gambiense* transmission cycle is mostly from human to human with an occasional involvement of an animal reservoir. The *T. b. rhodesiense* transmission cycle mainly involves wild and domestic animals, but intensified human-to-human transmission may occur during epidemics. There are no prophylactic drugs or vaccines available to prevent HAT and the few available treatments present a complex posology and severe side effects (Fevre et al., 2006; Brun et al., 2009). In 2011, less than 10,000 new cases were reported (Simarro et al., 2012), with many more cases probably remaining undetected as sleeping sickness occurs in remote rural areas. It is estimated that approximately 70 million people within tsetse-fly infested areas are at different levels of risk of contracting HAT, especially in countries as the Democratic Republic of Congo, Angola, South-Sudan, and the Central African Republic (Simarro et al., 2012; WHO, 2012). In addition to their importance in public health, at least seven other species of African trypanosomes cause severe disease in livestock known as African Animal Trypanosomiasis (AAT) or nagana. *T. vivax* and *T. congolense* are the major pathogens of cattle and other ruminants, while *T. simiae, T. godfreyi*, and *T. suis* causes high mortality in domestic pigs. Animal African Trypanosomiasis restricts agricultural development on the African continent despite the availability of prophylactic and curative drugs. Moreover, it is worrying to see drug effectiveness seriously threatened by an increasing resistance in animal trypanosomes (Delespaux et al., 2008).

Successful transmission is primarily the outcome of a crosstalk between the trypanosomes and the tsetse fly vector. This enables the parasite to undergo successive rounds of differentiation, proliferation, and migration in different locations of the fly, resulting in a final infective stage that is transmitted to a new mammalian host during each tsetse fly blood-feeding event. This Research Topic hosted in *Frontiers in Cellular and Infection Microbiology* focuses on the state-of-the-art on some key aspects of the fascinating biological interplay between African trypanosomes and the tsetse fly.

To prepare for life cycle progression, *T. brucei* parasites present in the bloodstream of an infected mammalian host have to generate stumpy forms that are pre-adapted to survive in the tsetse fly once they are taken up by the fly blood feeding. The molecular mechanisms that underpin the production of these stumpy forms and their signal perception pathways upon entry into the tsetse fly are detailed under the lights of recent discoveries in the review of Vidal et al., 2013. In the tsetse fly, trypanosomes have to go through specific developmental programs in order to survive and produce transmissible stage. Here, a mini-review will focus on the major advancements in our understanding of this trypanosome development in the tsetse fly (Rotureau and Van Den Abbeele, 2013). The different types of parasite cycle and the critical stages of the three epidemiologically most relevant trypanosome species, namely *T. vivax*, *T. congolense* and *T. brucei* will be compared. More emphasis is on *T. brucei* development since the parasite undergoes multiple morphological changes during the successive invasion of the tsetse alimentary tract to finally achieve the mammalian-infective stage in the salivary glands. Another review will attempt to elucidate how these morphological changes are possible for a parasite that has evolved a highly robust cell structure to survive the chemically and physically diverse environments in the fly (Ooi and Bastin, 2013).

During their journey in the tsetse fly, trypanosomes are challenged by a robust innate defense system. This is reviewed in the paper of Haines (Haines, 2013) with a focus on the tissues intimately associated with host defense. Moreover, the established symbiotic associations of tsetse flies with bacterial and viral microorganisms also modulate its vector competence for trypanosomes. A first review article will provide a detailed description of the tsetse symbiotic microbiome, and describes the physiology underlying host-symbiont and symbiont-symbiont interactions that occur in this fly (Wang et al., 2013). The diversity of the gut bacterial flora in the tsetse fly and the possible impact of newly identified bacteria to the vector competence will also be discussed in mini-review of Geiger et al. (2013).

In combination to the multiple trypanosome-tsetse crosstalk, environmental factors can also affect the parasite developmental barriers in the fly. Here, experimental work from Burkina Faso demonstrated that temperature stress could increase the susceptibility of tsetse to trypanosomes (Bouyer et al., 2013), thus opening the possibility to improve AAT risk mapping using satellite images.

Since African trypanosomes rely on tsetse flies for their transmission and dissemination, it is clear that a comprehensive knowledge on the multiple interactions between the parasite, the tsetse fly, its symbiotic flora, and the environment will allow for a better understanding of the transmission dynamics of these parasites in the natural context and could open new avenues for vector transmission control. In this respect, a change of paradigm has recently occurred since it is now recognized that vector control is an important part of the elimination strategy of *gambiense* HAT, as a complement to medical activities (Solano et al., 2013).
